# Presence of Epstein–Barr virus (EBV) antigens detected by sensitive methods has no influence on local immune environment in diffuse large B cell lymphoma

**DOI:** 10.1007/s00262-023-03617-x

**Published:** 2024-01-27

**Authors:** T. Mangiaterra, R. Alonso-Alonso, A. Rabinovich, M. De Dios Soler, L. Galluzzo, M. Soria, S. Colli, E. De Matteo, S. M. Rodriguez Pinilla, P. Chabay

**Affiliations:** 1 Molecular Biology Laboratory, Pathology Division, Multidisciplinary Institute for Investigation in Pediatric Pathologies (IMIPP), CONICET-GCBA, Ricardo Gutierrez Children’s Hospital, Buenos Aires, Argentina; 2 Pathology Department, Hospital Universitario Fundación Jiménez Díaz, Madrid, Spain; 3 Pathology Division, Marie Curie Hospital, Buenos Aires, Argentina; 4 Pathology Division, Prof. Dr. Juan P. Garrahan Hospital, Buenos Aires, Argentina; 5 Hematology Division, Ricardo Gutierrez Children’s Hospital, Buenos Aires, Argentina; 6 Pathology Division, Ricardo Gutierrez Children’s Hospital, Buenos Aires, Argentina

**Keywords:** EBV, DLBCL, LMP1, Traces, Microenvironment

## Abstract

EBV+ diffuse large B cell lymphoma (DLBCL) not otherwise specified (NOS) is a new entity confirmed by the World Health Organization (WHO) in 2017. In this new entity, the virus may contribute to a tolerogenic microenvironment. Traces of the virus have been described in DLBCL with more sensitive methods, in cases that were originally diagnosed as negative. The aim of this study was to analyze the expression of immune response genes in the tumor microenvironment to disclose the role of the virus and its traces in DLBCL. In 48 DLBCL cases, the expression of immune response genes and the presence of molecules that induce tolerance, such as *TIM3*, *LAG3* and *PDL1* by immunohistochemistry (IHC), were studied. To broaden the study of the microenvironment, tumor-associated macrophages (TMAs) were also explored. No significant differences were observed in the expression of immune response genes in the EBV+ DLBCL and those cases that were EBV− DLBCL but that exhibited viral traces, assessed by ViewRNA assay. Only the EBV+ DLBCL cases displayed a significantly higher increase in the expression of *CD8* and cytotoxic T cells detected by gene expression analysis, and of PDL1 in tumor cells and in the expression of CD68 in the tumor microenvironment detected by IHC, not observed in those cases with viral traces. The increase in CD8 and cytotoxic T cells, PDL1 and CD68 markers only in EBV+ DLBCL may indicate that traces of viral infection might not have influence in immune response markers.

**Supplementary Information:**

The online version contains supplementary material available at 10.1007/s00262-023-03617-x

## Introduction

Epstein Barr virus (EBV) is a human herpesvirus acquired in early life that establishes itself as a latent infection in memory B cells for life [1]. Its ability to transform the B cells has been associated with various specific lymphoma subtypes such as Burkitt, classic Hodgkin (cHL), plasmablastic, and diffuse large B cell lymphoma (DLBCL) [2]. Recently, in several EBV-associated B cell lymphomas, traces of EBV infection in cases originally considered as negative by EBERs in situ hybridization (ISH), the gold standard to detect EBV in lymphomas, were described. The presence of those traces provided the basis to explain the hit-and-run theory. According to this hypothesis, the viral infection in the B cell initially triggers the cellular transformation, and then, once the malignant process has been established, the lymphoma clone loses the EBV genome over time. Given the fact that the EBV traces may remain, some studies suggest that the virus could be involved in the pathogenesis of these lymphomas [3–5]. Recently, our group also proved the presence of traces of EBV in patients with DLBCL from Argentina, but suggested, since higher expression of the most important oncogenic protein, as well as increased viral load, was observed in cases with EBERs + cells by conventional ISH, that traces of EBV could not display a key role in DLBCL pathogenesis [6].

EBV+ DLBCL not otherwise specified (NOS) is a new entity confirmed by the World Health Organization (WHO) in 2017, with a high incidence in elderly patients, and at least 80% of the tumor cells positive for EBERs ISH [7]. In this new entity, it has been described that the virus may contribute to some alterations in the tumor microenvironment, such as the dysregulation of the programmed death ligand 1 (PDL1) [8].

The tumor microenvironment (TME) plays a key role in lymphomagenesis and is composed of T lymphocytes, macrophages, and natural killer (NK) cells, as well as stromal cells, blood vessels, and extracellular matrix (ECM) [9]. The expression of the molecules that induce tolerance in the TME of EBV+ DLBCL leads to an immune-tolerogenic microenvironment [10, 11]. The expression of PDL1, T cell immunoglobulin and mucin domain-containing protein-3 (TIM3), and lymphocyte activation gene-3 (LAG3) were described in DLBCL, as immune escape mechanisms to promote lymphomagenesis [12]. TIM3 and LAG3 are expressed in the surface of CD4 + and CD8 + T cells, to induce the impairment of the cell function, and, in consequence, to ultimately promote immune evasion. On the other hand, PDL1 can be expressed in tumor cells, as well as in the microenvironment, reducing immune cell function upon ligation to its ligand, PD1, expressed in T cells. Furthermore, it was described that PDL1 expression on the surface of tumor cells could be strongly associated with poor prognosis in patients with DLBCL [13, 14].

On the other hand, there are a few studies that explore tumor-associated macrophages (TMAs) in DLBCL. Macrophages are divided into at least M1 and M2 subtypes, depending on the expression of CD68 and CD163 markers, respectively, with opposing effector functions [15]. Riihijärvi. S et al. demonstrated a positive correlation between the protein and gene expression of macrophages markers in patients with DLBCL [16]. Furthermore, a high levels of CD68 protein and its RNA detected by gene expression approach in patients treated with chemoimmunotherapy were associated with favorable progression free survival (PFS) and overall survival (OS) in DLBCL [16, 17]. In contrast, a recent study described high levels of CD163 + M2 macrophages in EBV+ DLBCL, also associated with inferior outcomes [10].

Gene expression profiling (GEP) studies are used to quantitatively analyze factors related to the tumor and TME with prognosis and/or biological importance, and, specifically in DLBCL, it was used to classify distinct DLBCL molecular subtypes based on the cell of origin (COO) [18]. Furthermore, a customized platform that includes genes associated with immune response expressed by the different microenvironmental and neoplastic components was used to identify markers in peripheral T cell lymphoma (PTCL) and to recognize the expression of B cell genes for angioimmunoblastic T cell lymphoma (AITL). This approach was assessed in routinely processed paraffin-embedded samples, providing a more specific diagnosis and prognosis [19].

As described above, the role of TME is still under discussion in this new entity, EBV+ DLBCL, NOS, and it is still unexplored if the presence of EBV could trigger alterations at the TME, inducing a first “hit”, followed by the “run” after triggering, for example, a tolerogenic environment, as proposed by the hit-and-run theory. Under this hypothesis, once the alteration has been established, traces of EBV could be detected [5, 6]. In addition, in Argentina EBV-associated lymphomas have a higher incidence in children < 10 years, and almost 90% of patients are seropositive by the age of 3 years [20]. Furthermore, since our group previously suggested that traces of EBV could not display a key role in DLBCL pathogenesis, the aim of this study was to analyze the expression of immune response genes in the TME to disclose the role of the virus and its traces in DLBCL.

## Material and methods

### Patients and samples

A total of 48 DLBCL patients, previously characterized [6], were enrolled in this study, 26 pediatrics and 22 adults, collected retrospectively, based on the availability of sufficient material, from the archives at Pathology Division, at the Ricardo Gutierrez Children’s Hospital, and at the Marie Curie Hospital in Buenos Aires, Argentina. The samples were reviewed independently by 4 different pathologists: DDSM, GL, CS, and DME, according to the WHO classification for lymphoid neoplasms criteria [20]. The age range was 0–73 years (total median: 16 years, pediatrics median: 9 years, and adults: median 55 years). Institutional guidelines regarding human experimentation were followed, in accordance with the Helsinki Declaration of 1975. The Ricardo Gutierrez Children’s Hospital Ethics Committee (CEI) approved the study, and all the patients or patients’ guardians gave informed consent for the study.

### EBERs in situ hybridization (ISH)

EBERs in situ hybridization (ISH) was performed in formalin fixed paraffin-embedded (FFPE) tissue sections, using fluorescein isothiocyanate (FITC)-conjugated EBERs oligonucleotides as probes (Dako). A monoclonal antibody anti-FITC labeled with alkaline phosphatase was used to detect hybridized sites. In each hybridization run, an EBV-positive Hodgkin lymphoma FFPE paraffin-embedded tissue block was used as a control slide. A cutoff 20% of positive tumor cells we considered to define EBV+ DLBCL, NOS, was described [21].

### Double in situ hybridization

Double in situ hybridization with ViewRNA ISH Tissue 2-Plex Assay (Thermo Fisher, formerly Affymetrix) was performed in 36 FFPE DLBCL cases with good quality material, as previously reported [6]. Custom-specific probes sets against LMP1 (probe type 1) and EBNA2 (probe type 6) transcripts were customized by the manufacturer (Thermo Fisher, formerly Affymetrix), based on LMP1 and EBNA2 mRNA sequences. In order to define the presence of viral traces, the tumor cells that expressed LMP1 and/or EBNA2 transcripts in tumor cells were observed, and counted 100 × objective lenses in ten fields selected on the basis of the best-preserved tissue areas that contained positive cells, expressed per area as cells + /mm^2^.

### RNA extraction

Total RNA was isolated from FFPE DLBCL biopsies with RNeasy FFPE kit (Qiagen, Valencia, MA, EE. UU.), according to the manufacturer’s instructions, and quantified with NanoDrop 2000/2000C (Thermo Fisher Scientific, Waltham, MA, USA). The integrity and quality of the RNA were determined using TapeStation 4200 RNA ScreenTape kit (Agilent Technologies, Santa Clara, CA, USA), following the manufacturer’s instructions.

### nCounter gene expression assay

The Research Use Only Version of the NanoString XT assay along with the nCounter Flex Analysis System (NanoString Technologies, Seattle, WA, USA) was used to analyze gene expression. A customized 208 gene panel, listed in Supplementary Table 1, was used to evaluate gene expression. The gene panel included genes expressed by various components of the stroma and tumor cells in neoplastic diseases, as well as numerous genes known as therapeutic targets, in addition to a set of 8 housekeeping genes to normalize the values ​​of gene expression.

The probes were hybridized with 200 ng of total RNA for 16 h at 65 °C. The excess was removed, and immobilization of the probe transcription complexes was performed on a streptavidin-coated cartridge in the nCounter Preparation Station. Gene expression values ​​were normalized with housekeeping genes. Normalized count data were log2-transformed, and agglomerative hierarchical clustering of gene expression was performed. The data were analyzed using nSolver Analysis Software 4.0 (NanoString Technologies).

### Immunohistochemistry (IHC)

Antibodies against TIM3 (polyclonal, Abcam), LAG3 (clone [11E3], Abcam), CD68 (clone KP-1, Roche Ventana) and CD163 (clone MRQ-26, Roche Ventana) were used, as described [10, 14], to characterize microenvironment composition and to validate gene expression analysis. IHC detection of antibodies was carried out using a universal streptavidin–biotin complex-peroxidase detection system (UltraTek HRP Anti-Polyvalent Lab Pack, ScyTek, UT) according to the manufacturer’s instructions. Visualization of positive cells was performed using diaminobenzidine as chromogen. Appropriate positive controls were immunostained for each antibody. The counting of TIM3, LAG3, CD68 and CD163 positive cells was performed as follows: using the 100 × objective lenses and counting ten fields selected on the basis of the best-preserved tissue areas that contained immunopositive cells. The positive cells in tumor cells and in tumor-infiltrating lymphocytes (TILs) were counted, and the results were expressed as positive cells per area unit (cells + /mm2). Cells partly included in the fields were not counted.

### PDL1/PAX5 double staining

To differentiate the expression of PDL1 in tumor cells or at the microenvironment immunohistochemical double staining with PAX5 was carried out, to differentiate PDL1 + tumor cells (PAX5 +) from PDL1 + cells at the microenvironment (PAX5-), adapting the technique previously described [14]. 5-μm-thick FFPE tissue sections for all cases were used. Slides were incubated with anti-PAX5 (clone SP34, Dako) rabbit monoclonal antibody for 1 h at room temperature. PAX5 was detected with the Vectastain-ABC- Peroxidase kit, using diaminobenzidine (DAB) as chromogen. Then, the slides were incubated with a second primary antibody anti-PDL1 (clone 210,934, Abcam) diluted in 1/100 TBS-BSA 2%. Finally, the second primary antibody PDL1 was incubated overnight, and antibody signal amplification was performed using Alexa 594 anti-mouse, followed by Hoechst.

For double staining analysis, ImageJ imaging program was used. First, the positive cell counts of individual markers were made (PAX5 and PDL1). Then, pictures were merged and double-positive or single-positive cells were counted (PAX5 + PDL1 + and PAX5-PDL1 +). Afterward, positive cells were averaged and expressed per area unit (cells + /mm2). We defined double-positive (PAX5 + PDL1 +) for tumor cells expressing PDL1, PDL1t, and single-positive (PAX5-PDL1 +) for microenvironmental cells, PDL1m, in DLBCL, as described [8].

### Statistical analysis

Data were analyzed with GraphPad Prism5 (GraphPad Software Inc., San Diego, California, USA). Normality test was performed using Shapiro–Wilks’s test. Comparison of the means of cell counts by IHC was assessed by one-way ANOVA or Kruskal–Wallis tests for more than 2 groups, or by t test or Mann–Whitney, for 2 groups, based on normality test results between the groups according to EBV+ and EBV− cases, and cases with and without viral traces (positive or negative for LMP1 and/or EBNA2 transcripts). Outliers were defined using the robust test to compare data median absolute deviation (Mad) in Excel. For gene expression analysis, to test for statistical difference in medians, Mann–Whitney U tests were performed. To test if medians were different to zero, one-sample Wilcoxon signed rank tests were performed. Both types of tests were performed using wilcox.test function from the stats package in R [22]. *p* < 0.05 was considered statistically significant.

## Results

### Patients’ features and EBV analysis

Forty-eight DLBCL cases were included in this analysis. Twenty-six cases were pediatric and 22 were adults, and median age at diagnosis was 16 years. There was a male prevalence (52%), with 25 males and 23 females. On the basis of Hans’ IHC classification [23], 24 cases (50%) were classified as non-GC subtype, 20 (42%) as GC subtype, while in the remaining 4 cases (8%), the data were not available.

EBERs expression by ISH is summarized in Supplementary Table 2. Of the 48 DLBCL cases, 11 were positive, with ≥ 20% EBERs + cells (Supplementary Fig. 1A), as previously established in our population [21, 24]. The remaining 37 cases were negative by EBERs ISH, the gold standard to define EBV association with lymphomas.

Since traces of EBV infection were detected by sensitive methods in lymphomas [3, 4, 6], but its involvement in lymphomagenesis is still under discussion, double ISH to detect single copies of viral LMP1 and EBNA2 transcripts was assessed in 36 cases with good quality material. Of those 36 cases, 32 cases (89%) were defined as EBV− (< 20% EBERs + cells), whereas 4 cases (11%) were EBV+ (≥ 20% EBERs + cells) by ISH. In order to confirm the presence of traces, remarkably in the 32 EBV− cases, viral transcripts in tumor cells were detected in 17 cases (47%) (13 cases without EBERs expression), indicating the presence of viral traces (Supplementary Fig. 1 B and C). The presence of LMP1 transcripts, the most important viral oncogene, was confirmed in 6/13 cases positive for transcripts without EBERs expression by LMP1 immunohistochemical staining, as described [6].

According to those results, DLBCL cases were separated into different groups for further analysis. In order to evaluate the effect of traces of EBV infection in the expression of immune genes, initially, three groups were defined: i) EBV+ cases, with ≥ 20% EBERs + cells which also were positive for EBV transcripts, ii) EBV− Traces+ cases, including EBV− cases with < 20% EBERs + cells but expressing LMP1 and/or EBNA2 transcripts, and iii) Traces− cases, negative for both approaches. Given that only two groups are allowed to be compared by gene expression assay analysis, the three groups were clustered into two groups and mean expression was compared as follows: EBV+ (≥ 20% EBERs + cells) vs EBV− (< 20% EBERs + cells); Traces+ (positive for LMP1 and/or EBNA2 transcripts, regardless of EBV status by ISH) vs Traces− (negative for both transcripts); and EBV− Traces+ (cases that expressed viral LMP1 and/or EBNA2 transcripts but were EBV− by ISH) vs EBV− Traces− (cases that were negative by both methods). In contrast, specifically for microenvironment markers, the three initially defined groups were compared, in addition to a comparison between EBV+ vs EBV− cases to evaluate microenvironment composition with conventional EBERs cutoff [21].

### Nanostring XT nCounter gene expression assay in DLBCL

**Fig. 1:**
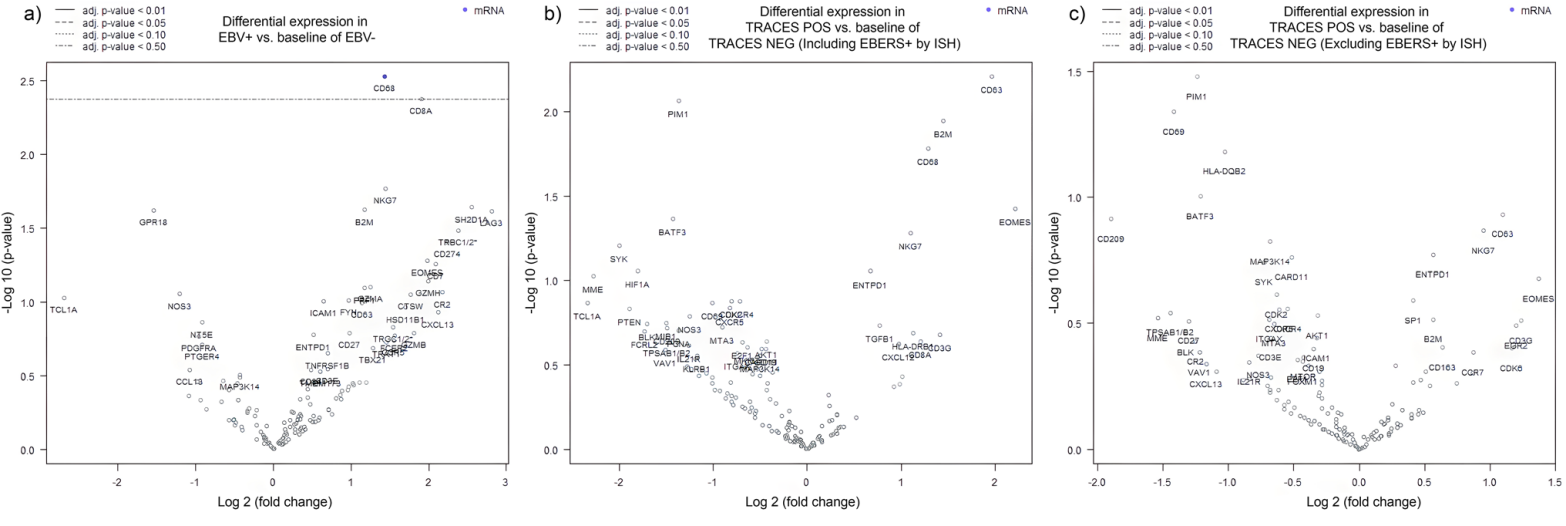
Volcano blots plot. The upregulated/unchanged/downregulated genes for (**a**) EBV+ vs EBV− DLBCL cases (**b**) DLBCL defined as Traces+ (positive for LMP1 and/or EBNA2 transcripts, including EBERs + by ISH) vs Traces− (negative for both transcripts). (**c**) EBV− Traces+ (excluding EBERs + by ISH) vs EBV− Traces−

**Fig. 2:**
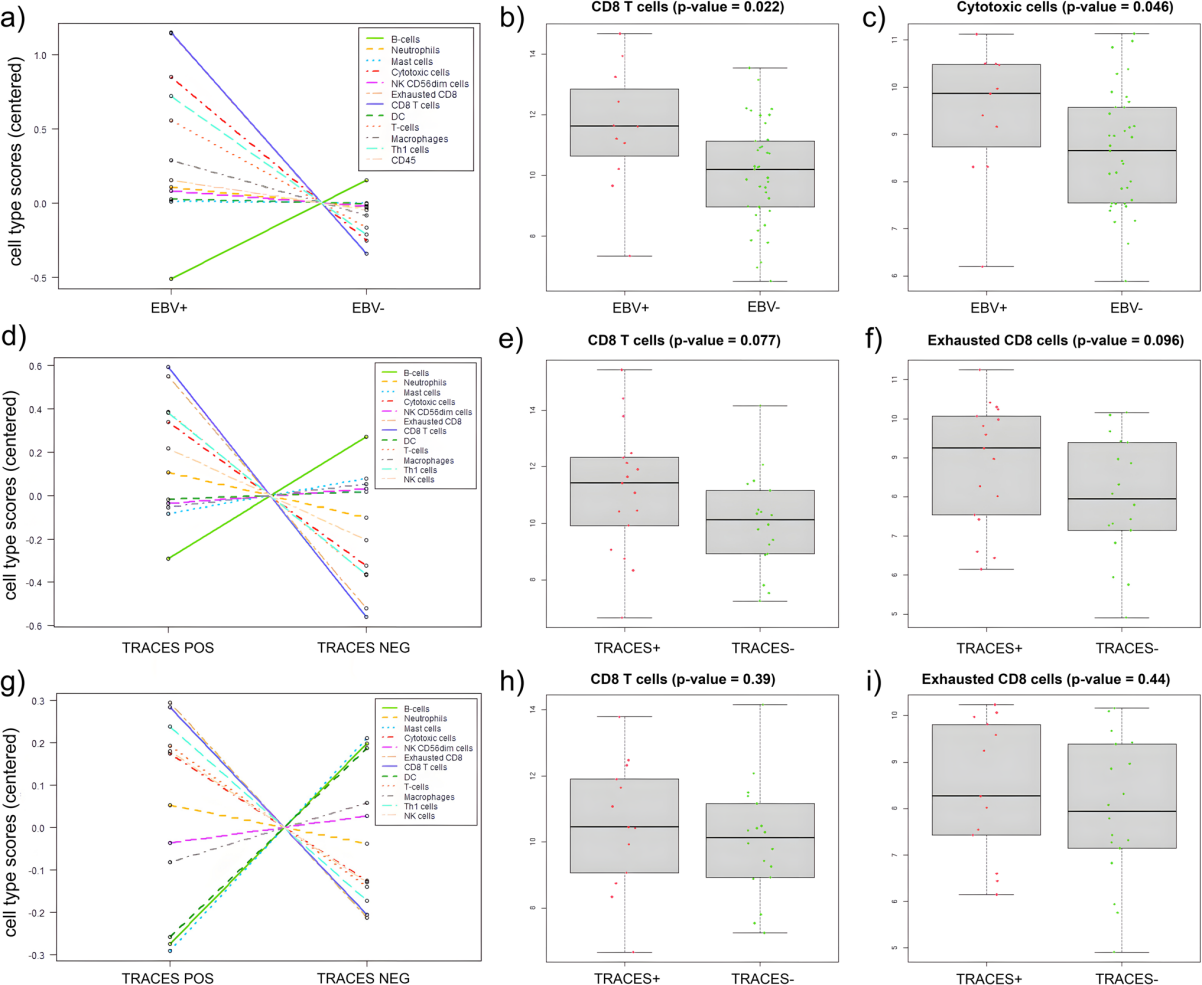
(**a**) Cell type profiling score vs. cell type measurement by covariate EBV+ vs EBV− DLBCL cases). (**b**) Box plot for CD8 T cells types between EBV+ vs EBV− DLBCL cases. (**c**) Box plot for cytotoxic cells between EBV+ vs EBV− DLBCL cases. (**d**) Cell type profiling score vs. cell type measurement by covariate Traces+ (including EBV+ cases) vs Traces−. (**e**) Box plot for CD8 T cells between Traces+ vs Traces− cases. (**f**) Box plot for exhausted CD8 T cells cell types between Traces+ vs Traces− cases. (**g**) Cell type profiling score vs. cell type measurement by covariate EBV− Traces+ vs EBV− Traces−. (**h**) Box plot for CD8 T cells between EBV− Traces+ vs EBV− Traces− cases. (**i**) Box plot for exhausted CD8 T cells cell types between EBV− Traces+ vs EBV− Traces− cases

The gene expression profile for immune response genes using customized NanoString Technologies platform in 48 DLBCL cases was assessed, in order to evaluate if traces of EBV infection could be involved in microenvironmental immune response. As previously mentioned, among those 48 DLBCL cases, 11 were considered EBV+ (according to ≥ 20% EBERs + cells used as cutoff) and 37 EBV− and included the cases with traces of EBV infection that were studied by double ISH for the detection of viral transcripts. As a first approach, the comparison in the expression of genes between the EBV+ vs EBV− cases was clustered as mentioned and analyzed (Fig. 1a). Even though significant differences in the gene expression between EBV+ and EBV− patients were not proved (FDR > 0.050 and log2 FC < 2), in the EBV+ cases, there was an increase in genes expression such as *CD8A*, *CD68*, *LAG3*, *CD274 (PDL1)*, among others. Furthermore, the EBV+ DLBCL cases displayed an enhanced gene expression for cell types such as CD8 T and cytotoxic cells (*p* = 0.022 and *p* = 0.046, respectively, Mann–Whitney U test) (Fig. 2a–c).

In contrast, when the gene expression was compared in the second cluster, between the cases with viral traces (including EBERs + cases) vs the cases without viral traces, the slight increase in gene expression like *LAG3* and *CD274 (PDL1)* previously observed for EBV+ vs EBV− cases was lost (Fig. 1b). The significant increase observed for gene expression signature for CD8 T cells in EBV+ cases shifted to a trend (*p* = 0.077, Mann–Whitney U test) in the cases that expressed viral transcripts. In addition, only a trend to an increase in exhausted CD8 T cells was observed in DLBCL cases with LMP1 and/or EBNA2 transcripts (Traces +) (*p* = 0.096, Mann–Whitney U test). (Fig. 2d–f). To exclude the influence of EBV+ cases by EBERs ISH, the gene expression for a third analysis, EBV− Traces+ vs EBV− Traces− cases, was assessed (Fig. 1c). As expected, the previous trend observed for CD8 T and exhausted CD8T cells was lost when EBV+ cases were excluded (*p* > 0.05, Mann–Whitney U test) (Fig. 2g–i).

### Tumor microenvironment (TME) in DLBCL

**Fig. 3:**
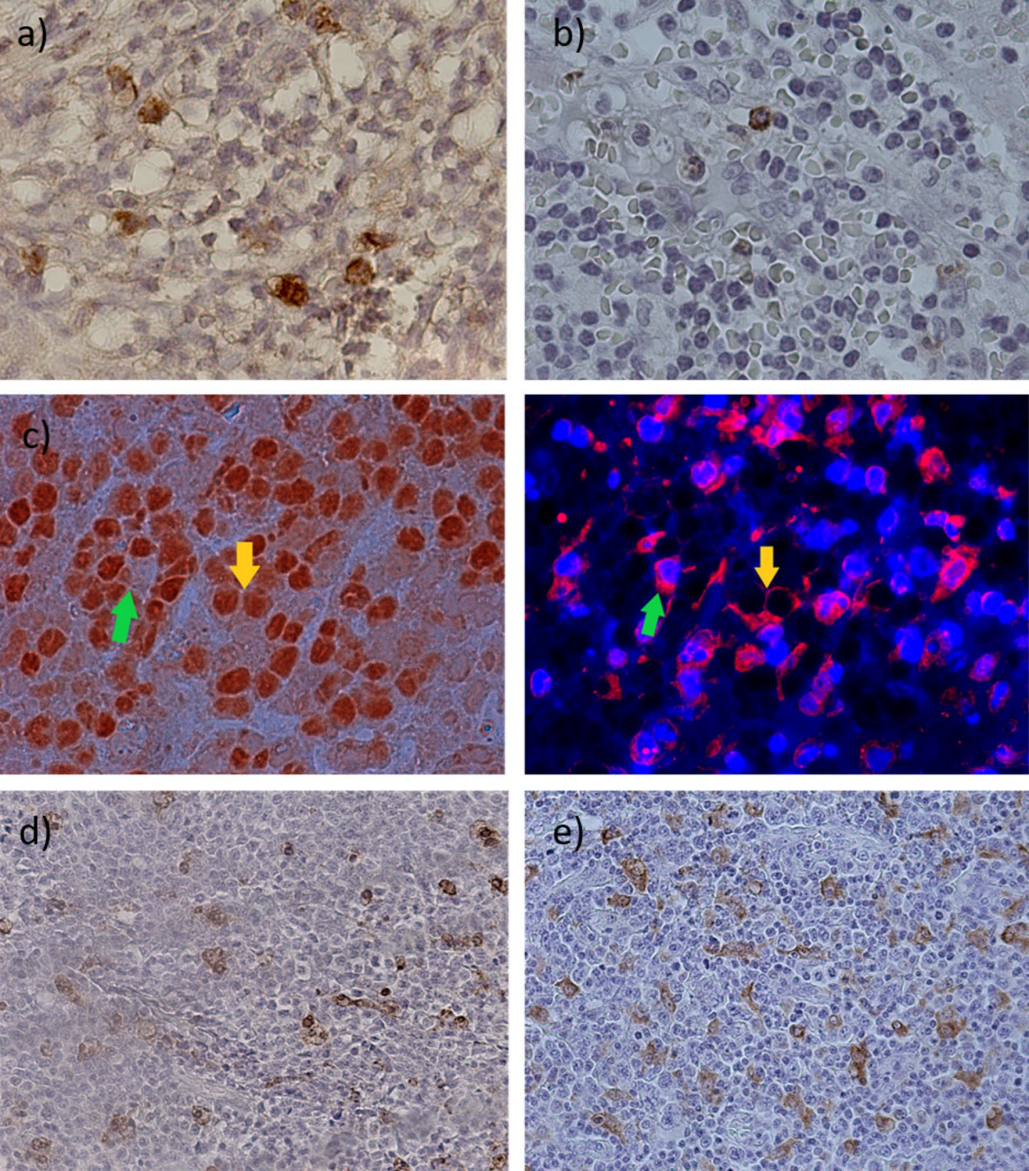
Immunohistochemical detection of tolerogenic markers. (**a**) Membranous positive staining of TIM3 in the microenvironment at ×1000, (**b**) Membranous positive staining of LAG3 in the microenvironment at ×1000, (**c**) Double stain for PAX5-PDL1 + (PDL1m) for cells in the microenvironment (green arrow) and PAX5 + PDL1 + (PDL1t) for tumor cells (yellow arrow) at ×1000, (**d**) Membranous positive staining of CD68 in the microenvironment at ×400, and (**e**) Membranous positive staining of CD163 in the microenvironment at ×400

**Fig. 4:**
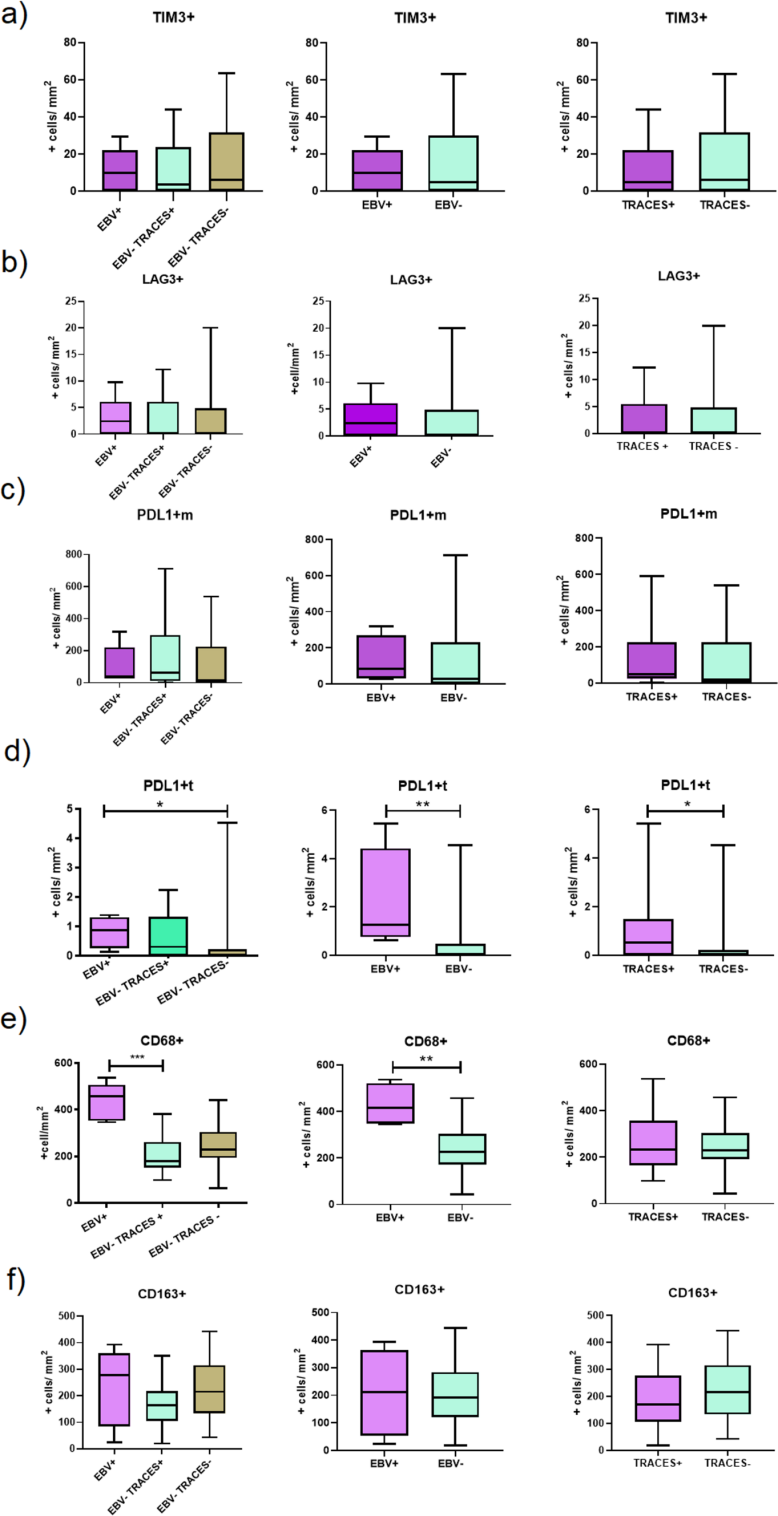
Tolerogenic markers in the three groups EBV+ , EBV− Traces+, and EBV− Traces−; between EBV+ vs EBV− cases, and between Traces+ (including EBV+ cases) vs Traces−: (**a**) TIM3 + cells in the microenvironment, (**b**) LAG3 + cells in the microenvironment, (**c**) PDL1 + m cells, (**d**) PDL1 + t cells, (**e**) CD68 + cells in the microenvironment, (**f**) CD163 + cells in the microenvironment

It was previously demonstrated, on the one hand, that EBV+ DLBCL may exhibit a tolerogenic microenvironment [10]. On the other hand, a slight increase in *PDL1* and *CD68* genes in EBV+ cases and a trend to a higher expression of exhausted cells in cases with LMP1 and EBNA2 transcripts (including EBV+ ones) were observed in this study. Therefore, several markers of tolerance, such as PDL1, TIM3, and LAG3, were evaluated in tumor cells and at the TME by single and double IHC in 47 DLBCL cases with available material for analysis, in order to confirm gene expression findings (Fig. 3a–e). Positive cell count was analyzed in relation to EBV presence and viral traces. For immunohistochemical analysis, at first three groups were compared: EBV+ (≥ 20% EBERs + cells), EBV− Traces+ (positive for LMP1 and/or EBNA2 transcripts), and Traces− (negative for both transcripts). No significant differences in the TIM3 + , LAG3 + , and PAX5-PDL1 + expression, that reflects protein expression PDL1 at the microenvironment (PDL1m), were observed among these three groups (*p* > 0.05; Kruskal Wallis, *p* > 0.05; Mann–Whitney) (Fig. 4, first column, a–c). Remarkably, when PDL1 protein expression in tumor cells (PAX5 + PDL1 + , PDL1 + t) was analyzed, a significant increase in PAX5 + PDL1 + cells was observed in EBV+ cases (*p* = 0.0024, Kruskal Wallis, *p* = 0.0120 Mann–Whitney) (Fig. 4d, first column).

Then, cases were grouped into EBV+ vs EBV− cases, and into Traces+ (including EBV+ cases) vs Traces− cases. No significant differences in TIM3 + , LAG3 + , and PDL1 + m expression were proved (*p* > 0.05, Mann–Whitney test) (Fig. 4a–c, second and third columns). However, PDL1 expression in tumor cells displayed significantly higher expression in both clustered scenarios (*p* = 0.0048, *p* = 0.0106; Mann–Whitney, respectively) (Fig. 4d, second and third columns), but no differences were proved when cases EBV− Traces+ were compared with EBV− Traces—(Fig. 4d, first column). Finally, since a slight increase in *CD68* gene expression was observed in EBV+ cases, and in order to explore the macrophage polarization markers, CD68 and CD163 were evaluated for each group defined. In addition, the polarization profile was defined by the CD68/ CD163 ratio in our cohort, as previously suggested [25]. Of the 47 cases available for immunohistochemical analysis, 57% showed an intermediate pattern (CD68/CD163 < 1.5), most of the EBV+ and EBV− Traces− cases (60% and 64%, respectively) and 34% showed M1 pattern (CD68/CD163 > 1.5), half of EBV− Traces+ cases (50%). Only 9% of DLBCL cases displayed the M2 polarization pattern (CD163/CD68 > 1.5). The CD68 + cell count was significantly higher in the EBV+ cases in comparison with EBV− Traces+ and EBV− Traces− cases (*p* = 0,0036; Kruskal Wallis, *p* = 0.0007, Mann–Whitney) (Fig. 4e, first column). Furthermore, when EBV+ vs EBV− cases were compared, there was also a significant increase in the former (*p* = 0.0034; Mann–Whitney) (Fig. 4e, second column). However, this difference was lost comparing Traces+ (including EBV+) vs Traces− cases (*p* > 0.05; Mann–Whitney) (Fig. 4e, third column). No differences were observed for CD163 + cell count (*p* > 0.05, Kruskal Wallis and Mann–Whitney test) (Fig. 4f).

## Discussion

Recently, it has been suggested that EBV may be involved in the pathogenesis of lymphomas in more cases than originally considered, based on the detection of viral traces sensitive techniques [3–5]. Furthermore, this hypothesis was also proposed for gastric cancer, where the methylation changes were studied as a mechanistic framework for how EBV can act in a hit-and-run manner [26]. However, even though EBV was detected by sensitive methods in patients with DLBCL from Argentina in more cases than originally considered, a higher expression of the most important oncogenic protein, LMP1, as well as increased viral load, was observed in cases with EBERs+ cells by conventional ISH, suggesting that traces of EBV could not display a key role in DLBCL pathogenesis [6]. Given the fact that EBV-associated lymphomas have a higher incidence in children < 10 years in Argentina, and almost 90% of patients are seropositive by the age of 3 years [20], our aim was to evaluate the local immune response in this population, to enlighten the role of traces of EBV infection in the pathogenesis of DLBCL.

Through the gene expression assay for immune response genes, no significant differences neither between the EBV+ groups compared to the EBV− cases nor between the cases that expressed viral traces compared to the cases without viral traces were demonstrated, in spite of the low number of patients included, one of the limits of this study. However, the slight increase in the expression of the *CD8A* gene prompted us to perform the analysis for cell types. Remarkably, the EBV+ DLBCL cases displayed an enhanced gene expression for cell types such as CD8 T and cytotoxic cells. This finding was not unexpected, since our group in a previous study showed an increase in CD8 + T cells and granzyme B + cytotoxic effector cells in EBV+ DLBCL NOS, associated with a tolerogenic microenvironment [27], that is the reason why in this study this increase was not confirmed by IHC. This increase in the expression of markers for CD8 T and cytotoxic cells for EBV+ cases but not for the cases that expressed viral traces might indicate the presence of traces could not be able to trigger this specific scenario. Alternatively, it could be assumed that an initial “hit” followed by the “run” is not sufficient to modify the local immune response, and the presence of high numbers of EBV-infected cells is required to induce cytotoxic response.

It is well known that the EBV+ DLBCL have a tolerogenic environment, characterized by the expression of PD1, TIM3, and LAG3 in tumor-infiltrating lymphocytes [10–14], and PDL1 in different types of cells, such as macrophages [14, 28, 29]. In contrast, the lack of significant differences in the expression of LAG3, TIM3, and PDL1 at the microenvironment may reveal that, specifically in this series, neither the EBV nor its traces could have influenced in the expression of these particular tolerogenic proteins at the tumor microenvironment.

The slight increase observed in *PDL1* gene expression *(CD274)* analyzed by Nanostring reflects its expression at the microenvironment, but also in tumor cells, another limit of this specific approach. In fact, increased PDL1 expression in tumor cells was related to EBV− presence in DLBCL [28, 30]. The expression of PDL1 in tumor cells could be consequence of PDL1 genetic alterations, which were demonstrated in EBV+ DLBCL [31], but also as a result of the activation by LMP1 and EBNA2 viral latent oncogenic proteins [32, 33]. In line with this, in the analyzed cohort, the expression of PDL1 only in tumor cells turned out to be significantly higher only for EBV+ cases, indicating that the virus, and its oncogenic proteins, could be involved in this increased expression. However, the traces of EBV infection might not be responsible for PDL1 upregulation.

The presence of different factors can induce macrophage polarization toward M1 or M2 profiles. M1, characterized by higher CD68 expression, is induced by a proinflammatory phenotype with tumoricidal activity. Instead, the M2 profile, with higher CD163 expression, promotes tissue repair and Th2 immune response and favors a tumorigenic propitious environment, promoting neoangiogenesis, tissue invasion, and metastasis [34]. In the context of EBV-associated lymphomas, M2 polarization was described in Burkitt lymphoma [35]. In contrast, M1 polarization was described in EBV-associated pediatric Hodgkin lymphoma [36, 37]. Concerning DLBCL, both EBV+ and EBV− cases exhibit a prevalence of M2 polarized macrophages so far [38, 39]. In contrast, in the whole series the intermediate polarization pattern confirmed by CD68/CD163 ratio < 1.5 predominated. Moreover, the small increase in CD68 gene expression observed in EBV+ DLBCL cases in our cohort was confirmed by immunohistochemical CD68 expression. Nevertheless, this increase was lost when only cases with traces of EBV infection were evaluated, indicating that they have no influence in CD68 upregulation.

In summary, this study provides further evidence of the role of traces of EBV infection in the pathogenesis of DLBCL, in a cohort from a population with a high incidence of EBV infection in children. Even though its detection may support the hit-and-run hypothesis, it seems like the immune response markers analyzed in this series, such as CD8, cytotoxic T cells, PDL1 and CD68, only are increased when EBERs is expressed in more than 20% of tumor cells, defined as EBV+ DLBCL. When only traces are detected by sensitive methods, they might not have influence in immune response markers, indicating that perhaps the initial “hit” is not enough to sustain the changes in the local milieu. Be as it may, further studies are needed to shed light on EBV involvement in the lymphomagenesis process in DLBCL.

### Supplementary Information

Below is the link to the electronic supplementary material.

Supplementary Fig. 1. A) EBV+ DLBCL case with ≥20% EBERs+ tumor cells at ×400. B) EBV- DLBCL case with viral LMP1 transcripts (red points) in tumor cells at ×1000. C) EBV- DLBCL case with viral EBNA2 transcripts (blue points) in tumor cells at ×1000 (PDF 10787 kb)

Supplementary file2 (DOCX 158 kb)

## Data Availability

The datasets generated during and/or analyzed during the current study are available from the corresponding author on reasonable request.
