# Supplementary figures and images for: Presence of Epstein–Barr virus (EBV) antigens detected by sensitive methods has no influence on local immune environment in diffuse large B cell lymphoma
Source: Cancer Immunol Immunother. 2024 Jan 27;73(2):29. doi: 10.1007/s00262-023-03617-x (PMC10821829; doi:10.1007/s00262-023-03617-x)

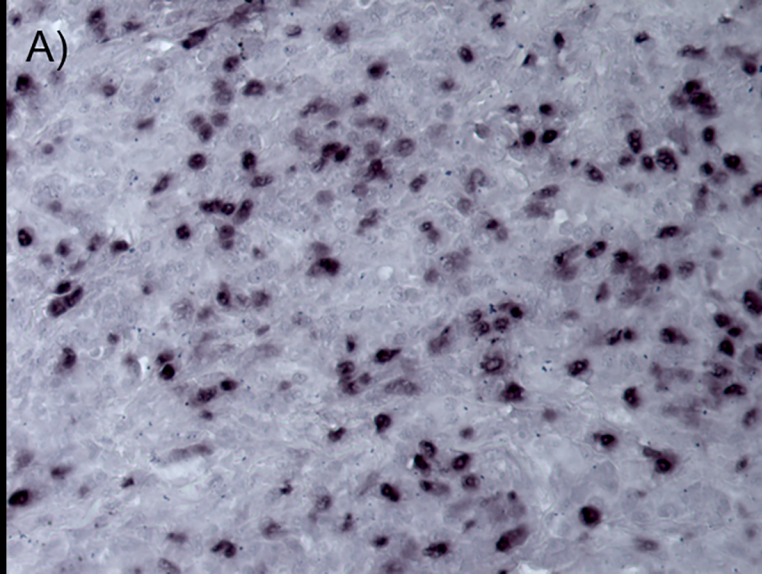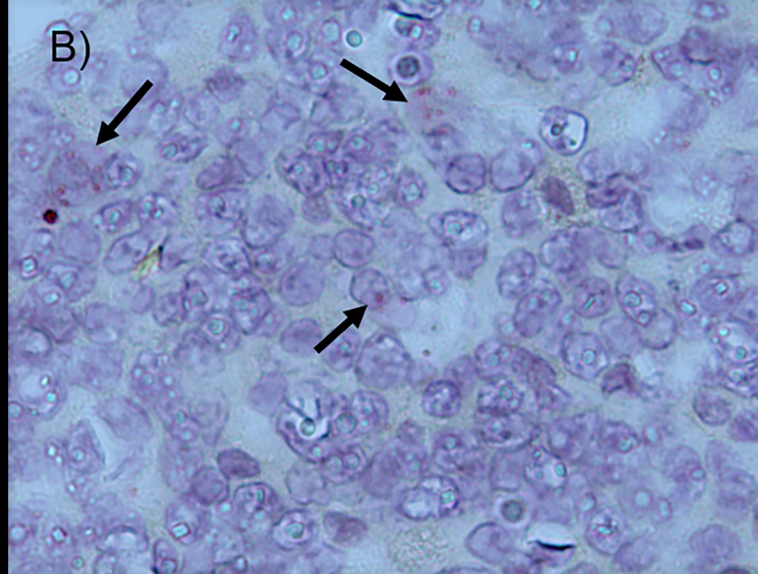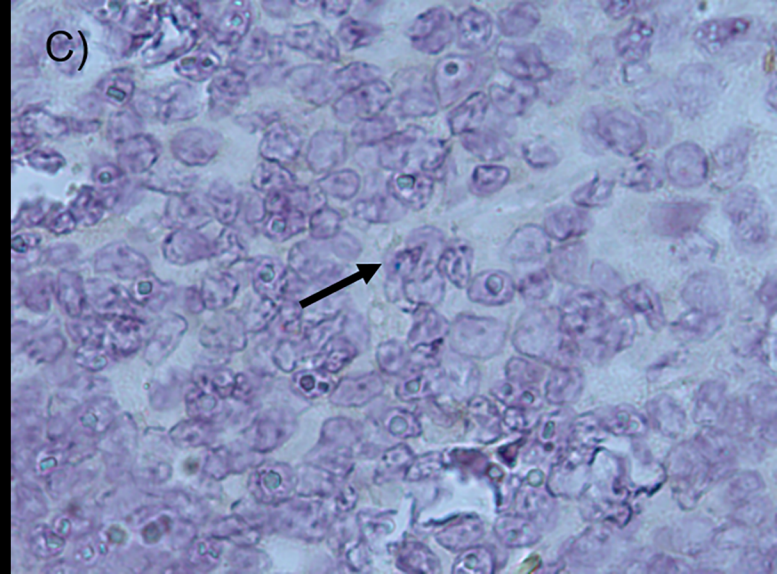

Supplement: Supplementary file 1 — Supplementary Fig. 1. A) EBV+ DLBCL case with ≥20% EBERs+ tumor cells at ×400. B) EBV- DLBCL case with viral LMP1 transcripts (red points) in tumor cells at ×1000. C) EBV- DLBCL case with viral EBNA2 transcripts (blue points) in tumor cells at ×1000 (PDF 10787 kb) [file 262_2023_3617_MOESM1_ESM.pdf]
