# Supplementary material for: Presence of Epstein–Barr virus (EBV) antigens detected by sensitive methods has no influence on local immune environment in diffuse large B cell lymphoma
Source: Cancer Immunol Immunother. 2024 Jan 27;73(2):29. doi: 10.1007/s00262-023-03617-x (PMC10821829; doi:10.1007/s00262-023-03617-x)
Supplement: Supplementary file 2 — Supplementary file2 (DOCX 158 kb) [file 262_2023_3617_MOESM2_ESM.docx]

***Supplementary Table 1****:* Customized genes NanoString panel used in the nCounter platform (NanoString Technologies, Seattle, WA, USA). The panel included 208 genes expressed by

the different components of the stroma and neoplastic cells, as well as genes known to be therapeutic targets. Gene expression values were normalized with respect to a set of eight housekeeping genes.

*
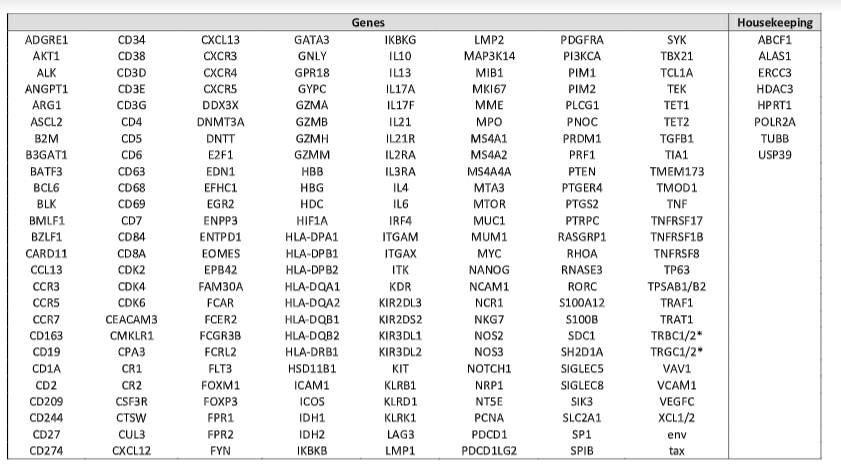
*

***Supplementary Table 2***. *Patients' features in relation to EBV status (n=48 cases DLBCL)*

|  | **EBV status cut off 20%** | |
| --- | --- | --- |
| **Patients` features** | **EBV+**  **EBERs +**  **tumor cells**  **≥ 20% (%)** | **EBV-**  **EBERs -**  **tumor cells**  **< 20%(%)** |
| **Age (years)** |  |  |
| Pediatrics < 18 | 8/26 | 18/26 |
| Adults ≥ 18 | 3/22 | 19/22 |
| **Gender** |  |  |
| Male | 6/25 | 19/25 |
| Female | 5/23 | 18/23 |
| **Histological subtype** |  |  |
| ABC | 5/24 | 19/24 |
| GC | 3/20 | 17/20 |
| ND | 2/4 | 2/4 |
